# Serotonergic Suppression of Mouse Prefrontal Circuits Implicated in Task Attention

**DOI:** 10.1523/ENEURO.0269-16.2016

**Published:** 2016-11-08

**Authors:** Michael K. Tian, Eric F. Schmidt, Evelyn K. Lambe

**Affiliations:** 1 Department of Physiology, University of Toronto, Toronto, ON, Canada; 2 Laboratory of Molecular Biology, Rockefeller University, New York, NY; 3 Department of Obstetrics and Gynecology, University of Toronto, Toronto, ON, Canada; 4 Department of Psychiatry, University of Toronto, Toronto, ON, Canada

**Keywords:** corticothalamic neurons, interneurons, medial prefrontal cortex, optogenetics, serotonin (5-HT)

## Abstract

Serotonin (5-HT) regulates attention by neurobiological mechanisms that are not well understood. Layer 6 (L6) pyramidal neurons of prefrontal cortex play an important role in attention and express 5-HT receptors, but the serotonergic modulation of this layer and its excitatory output is not known. Here, we performed whole-cell recordings and pharmacological manipulations in acute brain slices from wild-type and transgenic mice expressing either eGFP or eGFP-channelrhodopsin in prefrontal L6 pyramidal neurons. Excitatory circuits between L6 pyramidal neurons and L5 GABAergic interneurons, including a population of interneurons essential for task attention, were investigated using optogenetic techniques. Our experiments show that prefrontal L6 pyramidal neurons are subject to strong serotonergic inhibition and demonstrate direct 5-HT–sensitive connections between prefrontal L6 pyramidal neurons and two classes of L5 interneurons. This work helps to build a neurobiological framework to appreciate serotonergic disruption of task attention and yields insight into the disruptions of attention observed in psychiatric disorders with altered 5-HT receptors and signaling.

## Visual Abstract

**Figure:**
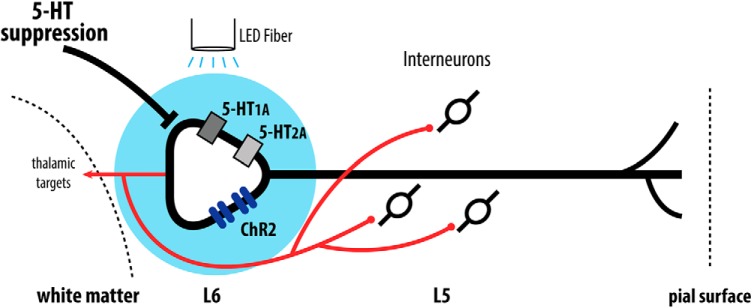

## Significance Statement

Although serotonin shapes and biases attention, the mechanisms underlying this phenomenon are not well understood. Layer 6 (L6) pyramidal neurons of medial prefrontal cortex play a critical role in performance on attention tasks, but their serotonergic modulation is unclear. Using electrophysiology and optogenetic techniques, we investigated the effects of serotonin on L6 pyramidal neurons and their local cortical circuits. We discovered a direct and serotonin-sensitive functional link from prefrontal L6 pyramidal neurons to GABAergic interneurons in L5, two populations of neurons essential for attention.

## Introduction

The medial prefrontal cortex (mPFC) is critical for “top-down” executive control of attention (Miller and Cohen, 2001; Knudsen, 2007), and disruption of its signaling impairs normal performance on attention tasks (Muir et al., 1996; Miner et al., 1997; Granon et al., 1998). Layer 6 (L6) of mPFC, in particular, plays an important role in attention (Alitto and Usrey, 2003; West et al., 2006; Zikopoulos and Barbas, 2006; Kassam et al., 2008; Bailey et al., 2010; Guillem et al., 2011) and is a major source of corticothalamic output (Guillery and Sherman, 2002; Thomson et al., 2002; Mercer et al., 2005; Watts and Thomson, 2005; West et al., 2006; Zikopoulos and Barbas, 2006; Parikh et al., 2007; Sherman, 2007; Kassam et al., 2008; Bailey et al., 2010; Thomson, 2010). Much less, however, is known about the cortico-cortical collaterals of L6 pyramidal neurons in mPFC. This question is increasingly urgent in view of recent work from primary sensory cortex showing that L6 pyramidal neurons send robust excitatory projections to fast-spiking cortical interneurons which achieve strong gain control over the cortical column (Olsen et al., 2012), together with recent work from mPFC showing that fast-spiking prefrontal interneurons are essential for attention (Kim et al., 2016). In mPFC, there remains much to be understood about the local targets of prefrontal L6 pyramidal neurons and the susceptibility of these attention circuits to neuromodulators such as serotonin.

Serotonin (5-HT) is known to shape and bias attention in human and nonhuman primates, with reduction of brain 5-HT enhancing attention (Schmitt et al., 2000; Gallagher et al., 2003; Booij et al., 2005; Wingen et al., 2007) and elevation of brain 5-HT impairing attention task performance (Riedel et al., 2005; Oranje et al., 2008; Watson et al., 2015). Yet the mechanisms underlying this relationship between 5-HT and attention are not well understood. As in primates, the deep layers of mPFC in rodents are well innervated by serotonin afferents (Wilson and Molliver, 1991; Linley et al., 2013; Goodfellow et al., 2014; Muzerelle et al., 2016). Therefore we can ask in a mouse model whether serotonin modulates prefrontal L6 pyramidal neurons known to play a role in task attention (Bailey et al., 2010; Guillem et al., 2011). In rodents, a substantial proportion of prefrontal L6 pyramidal neurons express 5-HT_1A_ and/or 5-HT_2A_ receptors (Chalmers and Watson, 1991; Pompeiano et al., 1992, 1994; Cornea-Hébert et al., 1999; Amargós-Bosch et al., 2004), with broad similarities in the expression of these 5-HT receptors in prefrontal L6 of human and nonhuman primates (de Almeida and Mengod, 2007, 2008; Mengod et al., 2015). Coexpression of 5-HT_1A_ and 5-HT_2A_ receptors is common in mPFC pyramidal neurons (Amargós-Bosch et al., 2004) and is observed in 48% of pyramidal neurons in L6 of mouse mPFC (Table 3 in Amargós-Bosch et al., 2004). Roles for 5-HT_1A_ and 5-HT_2A_ receptors in attentional performance have been suggested (Carli and Samanin, 2000; Koskinen et al., 2000; Winstanley et al., 2003; Wingen et al., 2007). Furthermore, the serotonergic system is dysregulated in several brain disorders that are accompanied by disruptions of attention, including autism (Chugani, 2002; Kane et al., 2012), schizophrenia (Luck and Gold, 2008), and mood disorders (Marvel and Paradiso, 2004; Jans et al., 2007; Murrough et al., 2011).

Here, we investigated how mPFC L6 pyramidal neurons are modulated by 5-HT, how L6 excitation affects the two major classes of L5 interneurons, and how 5-HT modulates these internal mPFC circuits. Our results reveal a strong 5-HT–elicited inhibition of L6 pyramidal neurons mediated by 5-HT_1A_ receptors and a lesser, state-dependent, and somewhat unexpected, contribution by 5-HT_2A_ receptors. We find that L6 pyramidal neurons robustly activate fast-spiking (FS) as well as non–fast-spiking (nFS) interneurons in L5. Finally, we show that these intracortical circuits in mPFC are strongly suppressed by 5-HT.

## Materials and Methods

### Experimental animals

We used BAC transgenic Swiss Webster mice with expression of eGFP driven by the synaptotagmin 6 promoter (Syt6-EGFP EL71, MMRRC; RRID:MMRRC 010557-UCD) made by the GENSAT Project (Gong et al., 2003). L6 pyramidal neurons in mPFC strongly express eGFP and facilitate visual targeting of these neurons for recording (Tian et al., 2014). Syt6 mice were kept heterozygous, and there were no significant differences in their 5-HT responses compared to their wild-type littermate controls or wild-type C57BL/6 mice (*F*_(2,64)_ = 0.58, *p* = 0.56, one-way ANOVA). To investigate the downstream synaptic connection of prefrontal L6 pyramidal neurons, we crossed GENSAT epiphycan BAC transgenic mice expressing Cre-recombinase (Epyc-Cre KR363, a gift from Dr. Nathaniel Heintz at Rockefeller University; RRID:MMRRC_036145-UCD) with Ai:32 mice (Jackson Laboratories, Bar Harbor, ME; RRID:IMSR_JAX:024109) to achieve eGFP-channelrhodpsin-2 expression in prefrontal L6 neurons (Epyc-ChR2). Wild-type littermates of the Epyc-ChR2 were used as controls to ensure that the UV light did not have effects in brain slices from mice lacking channelrhodopsin-2. Translating ribosome affinity purification (TRAP) and quantitative reverse-transcription (qRT)-PCR were used to confirm that Syt6 and Epyc-Cre neurons indeed represent an overlapping population of L6 glutamatergic neurons. All experimental animal procedures were performed in accordance with the University of Toronto and Rockefeller University Institutional Animal Care and Use Committee regulations.

### Electrophysiology

Coronal brain slices (400 µm) for electrophysiological recordings were obtained from adult male mice (postnatal 60 to 170 days; mean ± SEM; 101 ± 4 days; *n* = 41 mice). Brains were rapidly excised and chilled in 4°C oxygenated sucrose artificial cerebrospinal fluid (ACSF; 254 mm sucrose, 10 mm d-glucose, 24 mm NaHCO_3_, 2 mm CaCl_2_, 2 mm MgSO_4_, 3 mm KCl, 1.25 mm NaH_2_PO_4_; pH 7.4). Coronal slices (400 μm thick, 2.34–0.74 mm from bregma) were cut on a Dosaka Linear Slicer (SciMedia, Costa Mesa, CA) and recovered in 30°C oxygenated ACSF (128 mm NaCl, 10 mm d-glucose, 26 mm NaHCO_3_, 2 mm CaCl_2_, 2 mm MgSO_4_, 3 mm KCl, 1.25 mm NaH_2_PO_4_; pH 7.4) for at least 2 h.

Recovered slices were transferred to a perfusion chamber on the stage of a BX50W1 microscope (Olympus, Tokyo, Japan). ACSF was bubbled (95% O_2_, 5% CO_2_ at room temperature) and perfused the chamber at a rate of 3–4 ml/min. In addition to recording from L6 pyramidal neurons based on neuronal morphology and anatomical landmarks in wild-type mice, L6 in Syt6 mice was landmarked with fluorescently identified eGFP-positive neurons (X-cite Series 120; Lumen Dynamics, Mississauga, Canada; Tian et al., 2014). Recording electrodes (2–4 MΩ) containing 120 mm potassium gluconate, 5 mm KCl, 2 mm MgCl_2_, 4 mm K_2_-ATP, 0.4 mm Na_2_-GTP, 10 mm Na_2_-phosphocreatine, and 10 mm HEPES buffer (adjusted to pH 7.3 with KOH) were used to patch L6 pyramidal neurons. Interneurons in L5 were identified visually based on their unique morphology in infrared differential interference contrast (small, circular somata) in contrast to L5 pyramidal neurons (oriented, triangular shaped somata, relatively thick apical dendrites toward pia). A subset of patched interneurons was filled with Alexa Fluor 594 (20 µm) or Texas red dextran (0.15%) in the patch solution for morphological confirmation of these criteria. Interneurons were further subclassified as FS or nFS based on their electrophysiological spike pattern and maximal spike frequency. Multiphoton images were acquired with a Ti:sapphire laser (Mai Tai, Spectra-Physics, Fremont, CA) using an Olympus Fluoview FV1000 microscope and an Olympus XLPlan N 25× water-immersion objective. Neuronal membrane potential and holding current were recorded with an EPC10 (HEKA Electronik, Lambrecht/Pfalz, Germany) and corrected for the liquid junction potential (14 mV). All data were acquired at 20 kHz and low-pass filtered at 3 kHz with pClamp software (Molecular Devices, Palo Alto, CA). Threshold potentials for action potentials were detected using a derivative threshold of at least 20 mV/ms, and action potential amplitude was calculated as the change in membrane potential from threshold to the peak of the action potential. Intrinsic properties of L6 pyramidal neurons, as well as L5 FS and nFS interneurons, are summarized in Table 1.

**Table 1.** Intrinsic electrophysiological properties of three groups of neurons recorded: pyramidal neurons in L6, FS interneurons in L5, and nFS interneurons in L5.

	RMP (mV)	Input resistance (MΩ)	Spike amplitude (mV)	Peak firing frequency (Hz)	*n*
Layer 6 pyramidal neurons	–90.2 ± 0.6	128.5 ± 3.9	83.1 ± 0.6	22.4 ± 0.8	122
Layer 5 FS interneurons	–85.2 ± 1.2	114.3 ± 9.8	59.5 ± 2.2	109.8 ± 23.3	19
Layer 5 nFS interneurons	–82.7 ± 1.6	235.9 ± 30.0*	79.7 ± 1.9**	34.1 ± 3.0**	22

Neuronal properties shown are resting membrane potential (RMP), input resistance, spike amplitude, and peak firing frequency upon injection of a maximal suprathreshold current. Data are shown as mean ± SEM. Comparisons between L5 FS and nFS interneurons: *p < 0.05, **p < 0.001, unpaired *t* tests.

To examine the effects of 5-HT on L6 pyramidal neurons near rest and during spiking, we performed whole-cell patch-clamp recording in voltage clamp at –75 mV and in current clamp with current injections to elicit either constant spiking (2–3 Hz) at baseline or an initial membrane potential of –75 mV before depolarizing current injections (1 s, 25-pA steps, 15-s intervals) were used to assess input–output relationships. For the latter experiment, the frequency of action potential firing was measured for each depolarizing current step and plotted against the magnitude of the injected current step.

### Pharmacology

Acute responses to 5-HT were probed by bath application of 5-HT (serotonin creatinine sulfate, Sigma-Aldrich, St. Louis, MO; 10 µm; 30 s) in ACSF. To examine the effect of 5-HT on the excitability of L6 pyramidal neurons, 5-HT (10 µm) was bath applied until a steady-state response was reached, and remained in bath throughout the duration of the input–output test protocols (∼2 min total application). Selective antagonists and agonists were from Tocris (Bristol, UK), except where mentioned. Antagonists for 5-HT_1A_ receptors (30 nm WAY100635, 10 µm NAN-190) and 5-HT_2A_ receptors (30 nm MDL100907; 2 µm ketanserin; 300 nm to 3 µm ritanserin) were applied in bath for 10 min before further experiments with 5-HT. There were no significant differences between effects of 300 nm and 3 µm ritanserin, and results were grouped for analysis. TCB-2 was used as a specific agonist of 5-HT_2A_ receptors (300 nm to 1 µm). Other agonists and antagonists used for characterization of the 5-HT response in L6 neurons were as follows: 2 µm tetrodotoxin (TTX) (Alomone Labs, Jerusalem, Israel), 20 µm 6-cyano-7-nitroquinoxaline-2,3-dione, 50 µm d-2-amino-5-phosphonovaleric acid, 100 µm picrotoxin, 1 µm CGP52432, and 10 µm 8-OH-DPAT.

### Optogenetic stimulation

Channelrhodopsin-expressing neurons in Epyc-ChR2 mice were stimulated by blue LED light (473 nm) delivered by optic fiber (Thorlabs, Newton, NJ) mounted on a mechanical micromanipulator (Narishige International, East Meadow, NY). Light stimulation was directed directly to L6 of mPFC by targeted positioning of the optic fiber. Twenty light pulses (2–5 ms each) were delivered at 20 Hz to stimulate L6 neurons. This stimulation profile was sufficient to elicit robust activation of L6 pyramidal neurons expressing channelrhodopsin. In control experiments with brain slices from littermate mice lacking channelrhodopsin, light stimulation did not elicit a response in either L6 pyramidal neurons or L5 interneurons. Responses to light stimulation in L6 pyramidal neurons and L5 interneurons were measured in current-clamp from a baseline membrane potential of –75 mV held by continuous injection of depolarizing current. Response latency in L6 pyramidal neurons expressing channelrhodopsin was calculated from the time of light-on to the onset of the corresponding membrane potential change. Time-to-spike for L6 neurons from light-on was also calculated using the peak of the first resulting action potential. In L5 interneurons, the latency to response from L6 activation was calculated in voltage clamp as the time taken from light-on to the onset of the postsynaptic current, then corrected by the time-to-spike in L6 pyramidal neurons. Pairwise analysis of the effects of 5-HT on the excitation of L5 interneurons by optogenetic activation of L6 were performed using light stimulus that was able to elicit at least four action potentials in patched L5 interneurons. Light stimulus intensity to elicit a baseline of at least four action potentials did not differ between FS and nFS interneurons (*t*_14_ = 1.4, *p* = 0.18, unpaired *t* test).

### Statistical analysis

All recordings were analyzed using Clampfit software (Molecular Devices). Statistical analyses were performed with GraphPad Prism (GraphPad Software, La Jolla, CA). Analyses performed were one-sample *t* test, unpaired Student’s *t* test, paired Student’s *t* test, one-way ANOVA, two-way ANOVA, and two-way repeated-measures ANOVA. All tests were two-sided. Dunnett’s multiple comparison tests were performed *post hoc* to compare changes in action potential firing in L6 neurons elicited by 5-HT. Sidak’s multiple comparison tests were used to compare differences in spike frequency at individual injected current steps in the presence of 5-HT. All data are presented as mean ± SEM.

### Translating ribosome affinity purification and quantitative RT-PCR

Adult (8–12 weeks old) Epyc-Cre mice under ketamine/xylazine (100/10 mg/kg) anesthesia received single bilateral stereotaxic injections of 0.25 µl AAV-FLEX-EGFPL10a virus (3.75 × 10^12^ genome copies/ml) into the mPFC (1.54 AP from bregma, 0.4 ML, –1.80 DV from dura). Animals were sacrificed in a controlled CO_2_ chamber 3 weeks after surgery, brains were rapidly dissected in ice-cold Hanks balanced salt solution containing 2.5 mm HEPES-KOH (pH 7.4), 35 mm glucose, 4 mm NaHCO_3_, and 100 µg/ml cycloheximide. The cortex was isolated from the rest of the brain, and each hemisphere was split along the coronal plane at the level of the genu of the corpus callosum (∼1.6 mm AP from bregma). The rostral portion was saved as the “PFC” and used for TRAP. Tissue from three mice (male and female) was pooled for each sample, and three biological replicates were collected. Polysome immunoprecipitations (IPs) were carried out as previously described (Schmidt et al., 2012; Heiman et al., 2014). Briefly, the tissue was homogenized in extraction buffer containing 10 mm HEPES-KOH (pH 7.4), 150 mm KCl, 5 mm MgCl_2_, 0.5 mm dithiothreitol, 100 µg/ml cycloheximide, RNasin (Promega, Madison, WI), and SUPERas-In (Invitrogen, San Diego, CA) RNase inhibitors, and complete EDTA-free protease inhibitors (Roche, Basel, Switzerland), and then cleared by centrifugation at 2000 × *g*. IGEPAL CA-630 (NP-40, Sigma-Aldrich) and diheptanoyl phosphatidylcholine (Avanti Polar Lipids, Alabaster, AL) were both added to the S2 supernatant for a final concentration of 1% each, followed by centrifugation at 20,000 × *g*. Polysomes were immunoprecipitated from the S20 supernatant using 100 µg monoclonal anti-EGFP antibodies (50 µg each of clones 19C8 and 19F7; Heiman et al., 2008) bound to biotinylated protein L (Pierce, Thermo Fisher, Waltham, MA) coated streptavidin-conjugated magnetic beads (Invitrogen), and washed in high salt buffer containing 10 mm HEPES-KOH (pH 7.4), 350 mm KCl, 5 mm MgCl_2_, 1% IGEPAL CA-630, 0.5 mm dithiothreitol, 100 µg/ml cycloheximide, and RNasin RNase inhibitors (Promega). IPs were carried out overnight at 4°C. Bound RNA was purified using the Absolutely RNA Nanoprep kit (Agilent, Santa Clara, CA). RNA was also purified from the pre-IP supernatant to serve as whole-PFC “input” samples. RNA quantity was measured with a Nanodrop 1000 spectrophotometer, and quality was assayed on an Agilent 2100 Bioanalyzer. Only samples with RNA integrity values >7.0 were used for qRT-PCR analysis. cDNA was synthesized from 15 ng of IP or input total RNA using the Ovation qPCR System (NuGEN Technologies, Carlos, CA) following the manufacturer’s protocol. qRT-PCR was performed on an Applied Biosystems StepOnePlus Fast Real-Time PCR System using commercially available Taqman assays (Table 2) and following standard cycling conditions (50°C for 2 min, 95°C for 10 min, then 40 cycles of 95°C for 15 s and 60°C for 1 min). Ten nanograms of cDNA was used for each qRT-PCR reaction, and technical triplicates were run for each of the biological triplicates from TRAP IP and input samples. The mean C_T_ for technical replicates was used for quantification. Data were normalized to *Gapdh* by the comparative C_T_ (2^–ΔΔCT^) method (Livak and Schmittgen, 2001). Data are presented as mean ± SEM of biological triplicates. Statistical significance was calculated between the normalized expression values (2^–ΔCT^) from the IP and input biological replicates for each gene by Student’s *t* test in Microsoft Excel.

**Table 2.** TaqMan gene expression assay.

Symbol	Gene name	Assay	Dye
*Aldh1l1*	Aldehyde dehydrogenase 1 family, member L1	Mm03048957_m1	FAM
*Cnp*	Cyclic nucleotide phosphodiesterase 1	Mm01306640_m1	FAM
*Foxp2*	Forkhead box P2	Mm00475030_m1	FAM
*Gad1*	Glutamic acid decarboxylase 1	Mm00725661_s1	FAM
*Gapdh*	Glyceraldehyde-3-phosphate dehydrogenase	Mm99999915_g1	FAM
*Htr1a*	5-Hydroxytryptamine (serotonin) receptor 1A	Mm00434106_s1	FAM
*Htr2a*	5-Hydroxytryptamine (serotonin) receptor 2A	Mm00555764_m1	FAM
*Ntsr1*	Neurotensin receptor 1	Mm00444459_m1	FAM
*Slc17a7*	Vesicular glutamate transporter 1	Mm00812886_m1	FAM
*Syt6*	Synaptotagmin VI	Mm01308768_m1	FAM

## Results

### Serotonin robustly inhibits L6 pyramidal neurons of mPFC

**Figure 1.**
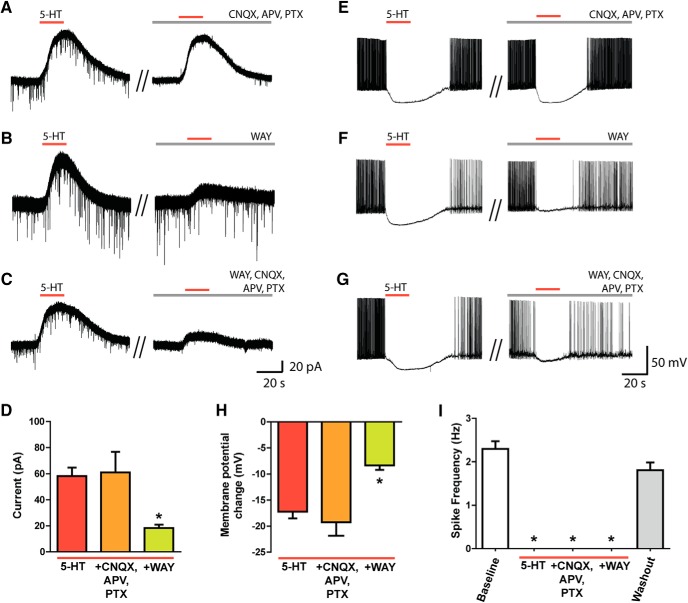
5-HT inhibits L6 pyramidal neurons of medial prefrontal cortex. Responses to 5-HT were probed in voltage-clamp and current-clamp by bath application of 5-HT. Representative voltage-clamp traces of the 5-HT response in L6 pyramidal neurons show that responses to 5-HT are stable and persist in the presence of synaptic blockers (***A***), are significantly suppressed by WAY100635 (***B***), and are similarly suppressed by a combination of WAY100635 and synaptic blockers (***C***). ***D***, 5-HT elicits strong outward currents on L6 pyramidal neurons of mPFC (*n* = 28) that can be pharmacologically modulated (*F*_(2,53)_ = 11.8, *p* < 0.0001, one-way ANOVA). *Post hoc* analyses show that these currents persist in the presence of synaptic blockers (*q* = 0.2, *p* > 0.05, *n* = 7, Dunnett’s multiple comparison test) but are significantly suppressed by WAY100635 (*q* = 4.6, *p* < 0.0001, *n* = 19). Responses to 5-HT were probed in current-clamp in L6 pyramidal neurons of medial prefrontal cortex with current injection to elicit steady firing (∼2–3 Hz) at baseline. Representative current-clamp traces show that responses to 5-HT are inhibitory, repeatable, and unaffected by synaptic blockers (***E***) and not fully blocked by WAY100635 (***F***) or by WAY100635 and synaptic blockers (***G***). ***H***, L6 pyramidal neurons of mPFC are strongly hyperpolarized by 5-HT (*n* = 26). *Post hoc* analyses of pharmacological effects on this hyperpolarization (*F*_(2,50)_ = 17, *p* < 0.0001, one-way ANOVA) show that this inhibition is unaffected by synaptic blockers (*q* = 0.8, *p* > 0.5, *n* = 6, Dunnett’s multiple comparison test) but greatly reduced by WAY100635 (*q* = 5.4, *p* < 0.0001, *n* = 21). ***I***, Action potential firing was significantly affected by 5-HT (*F*_(4,103)_ = 37, *p* < 0.0001, one-way ANOVA). *Post hoc* analyses reveal that baseline firing was strongly suppressed by 5-HT (*q* = 9, *p* < 0.0001, *n* = 17, Dunnett’s multiple comparison test) and remained suppressed by 5-HT in synaptic blockers (*q* = 6.1, *p* < 0.0001, *n* = 6). The suppression was not blocked by WAY100635 (*q* = 8.8, *p* < 0.0001, *n* = 16) and returned to baseline levels after washout of 5-HT (*q* = 2.3, *p* > 0.05, *n* = 36).

Here, we investigated the electrophysiological consequences of 5-HT on pyramidal neurons in L6 of mPFC. Experiments in voltage clamp showed robust and replicable outward currents (58.3 ± 6.4 pA, *n* = 28; Fig. 1*A*, *B*) in response to bath application of 5-HT (10 µm, 30 s). These 5-HT–elicited currents were dose-dependent, with an EC_50_ of 5.7 ± 0.1 µm (*n* = 7, *r* ^2^ = 0.9). The lack of significant change in these responses to blocking voltage-gated sodium channels with TTX (2 µm, 10 min in bath; *t*_3_ = 1.2, *p* = 0.3, *n* = 4, paired *t* test), to blocking AMPA, NMDA, and GABA-A receptors (6-cyano-7-nitroquinoxaline-2,3-dione [20 µm], 2-amino-5-phosphonovaleric acid [50 µm], picrotoxin [100 µm], *t*_6_ = 0.5, *p* = 0.6, *n* = 7, paired *t* test; Fig. 1*A*, *D*), as well as to blocking these synaptic receptors together with GABA-B blockade with CGP52432 (10 µm; *t*_3_ = 0.27, *p* = 0.8, *n* = 4, paired *t* test) suggest direct mediation by 5-HT receptors on L6 pyramidal neurons themselves. We found that the specific 5-HT_1A_ antagonist WAY100635 (30 nm, 10 min in bath) significantly reduced the 5-HT–mediated current in L6 pyramidal neurons (∼70% reduction to 18.3 ± 2.6 pA, *t*_18_ = 4.9, *p* < 0.0001, *n* = 19, unpaired *t* test; Fig. 1*B–D*). The 5-HT_1A_ agonist 8-OH-DPAT (10 µm) elicited outward currents of similar magnitude to the 5-HT current in L6 pyramidal neurons (5-HT, 61.2 ± 12.8 pA; 8-OH-DPAT, 48.5 ± 11.1 pA, *t*_5_ = 1.6, *p* = 0.2, *n* = 6, paired *t* test).

To investigate the functional effects of 5-HT on L6 pyramidal neurons during excitation, we used current-clamp and bath-applied 5-HT in the presence of injected positive depolarizing current sufficient to elicit action potential firing (2–3 Hz). Under these conditions, 5-HT hyperpolarized L6 neurons (–16.3 ± 1.6 mV, *n* = 17) and fully and significantly inhibited action potential firing in every recorded neuron (*t*_7_ = 13.5, *p* < 0.0001, *n* = 8, paired *t* test; Fig. 1E, H, I). This suppression was repeatable in the same neuron after washout and was not affected by the presence of synaptic blockers (Fig. 1E, H, I). Antagonism of 5-HT_1A_ receptors by WAY100635 significantly reduced the 5-HT–mediated hyperpolarization (–7.8 ± 0.7 mV, *t*_18_ = 5.0, *p* < 0.0001, *n* = 19, unpaired *t* test; Fig. 1F–I). Unexpectedly, however, 5-HT still robustly and significantly inhibited action potential firing in every neuron (*t*_13_ = 12, *p* < 0.0001, *n* = 14, paired *t* test; Fig. 1F–I). This strong and significant suppression of L6 spiking by 5-HT was also observed in the presence of synaptic blockers (*t*_3_ = 4.8, *p* = 0.02, *n* = 4, paired *t* test). These data show a robust and repeatable 5-HT inhibition of L6 neurons by 5-HT with a component mediated by 5-HT_1A_ receptors. However, the continued suppression of action potential firing by 5-HT after blockade of 5-HT_1A_ receptors suggests the involvement of an additional 5-HT–mediated mechanism for inhibition of mPFC L6 pyramidal neurons.

### Serotonergic 5-HT1A and 5-HT2A receptors cooperate in inhibiting L6 pyramidal neurons

**Figure 2.**
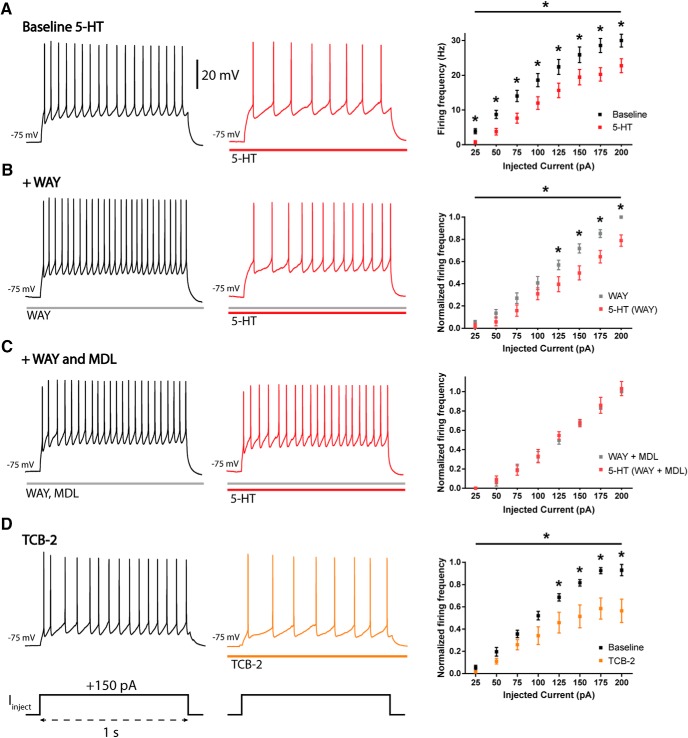
Combined activation of serotonergic 5-HT_1A_ and 5-HT_2A_ receptors mediate inhibition of L6 neuronal excitability at suprathreshold potentials. Incremental current steps were injected into patched L6 pyramidal neurons, and their output in firing frequency was measured. Shown are representative recordings of the response to a 150-pA current step in single L6 pyramidal neurons (left) and the response to the same 150-pA current step in the presence of 5-HT (middle). The input–output relationship for each group is plotted (right). ***A***, The input–output relationship of L6 pyramidal neurons is significantly right-shifted by 5-HT (*F*_(1,168)_ = 31, *p* < 0.0001, repeated-measures two-way ANOVA). *Post hoc* analysis showed significantly fewer elicited action potentials at every input step (*p* < 0.05, Sidak’s multiple comparisons test). ***B***, L6 excitability is significantly suppressed by 5-HT in the presence of WAY100635 (*p* < 0.0001, *F*_(1,72)_ = 72, repeated-measures two-way ANOVA), an effect especially prominent at higher input steps (125- to 200-pA steps, *p* < 0.05, Sidak’s multiple comparisons test). ***C***, L6 suppression by 5-HT is fully blocked by simultaneous blockade of both 5-HT_1A_ and 5-HT_2A_ receptors by specific antagonists WAY100635 and MDL100907 (*F*_(1,32)_ = 0.8, *p* = 0.4, repeated-measures two-way ANOVA). ***D***, TCB-2, a selective 5-HT_2A_ receptor agonist, inhibits L6 neuronal firing (*F*_(1,80)_ = 24, *p* < 0.0001, repeated-measures two-way ANOVA), also more prominently at higher input steps (125- to 200-pA steps, *p* < 0.05, Sidak’s multiple comparisons test).

To interrogate this unidentified component of 5-HT inhibition of L6 pyramidal neurons, action potentials were elicited by a series of incremental square depolarizing pulses before and during 5-HT application. Despite the stronger activation, 5-HT still significantly reduced spike frequency at each injected current step, as shown by the significant right-shift in the input–output curve (inhibitory effect of 5-HT: *F*_(1,168)_ = 31, *p* < 0.0001, *n* = 22, repeated measures two-way ANOVA; Fig. 2*A*). *Post hoc* tests show that significantly fewer action potentials were elicited by input current in 5-HT compared with baseline at every step (*p* < 0.05, Sidak’s multiple comparisons test). Of note, the 25-pA step was suprathreshold for 17 of 22 neurons at baseline and only 3 of 22 neurons in 5-HT (*p* < 0.0001, Fisher’s exact test). Spiking was significantly restored after a 5-min washout of 5-HT (*F*_(1,168)_ = 28, *p* < 0.0001, repeated-measures two-way ANOVA). Activation of 5-HT_1A_ receptors by 8-OH-DPAT also significantly suppressed firing of L6 neurons (50-pA current injection, baseline, 4.0 ± 1.1 Hz; 8-OH-DPAT, 0.8 ± 0.6 Hz; *t*_5_ = 5.3, *p* = 0.003, *n* = 6, paired *t* test), as did 8-OH-DPAT across a range of depolarizing steps (*F*_(5,12)_ = 35.7, *p* < 0.0001, two-way repeated measures ANOVA, data not shown).

Yet, further experiments suggest that 5-HT recruits an additional receptor beyond 5-HT_1A_ to inhibit the excitability of L6 pyramidal neurons. Significant 5-HT suppression of L6 neuronal excitability continued after antagonism of 5-HT_1A_ receptors by WAY100635, with a significantly right-shifted input–output curve (inhibitory effect of 5-HT in WAY100635: *F*_(1,72)_ = 72, *p* < 0.0001, *n* = 10, repeated measures two-way ANOVA; Fig. 2*B*). Consistent with our above data, this result suggests the participation of at least one additional subtype of 5-HT receptor in inhibiting L6 pyramidal neurons. The 5-HT_2A_ receptors that are coexpressed with 5-HT_1A_ receptors in 48% of L6 pyramidal neurons in mouse mPFC (Table 3 in Amargós-Bosch et al., 2004) are an unusual candidate to underlie the 5-HT–mediated suprathreshold suppression of spiking. These receptors typically recruit excitatory effectors (Lambe and Aghajanian, 2001; Zhang and Arsenault, 2005; Weisstaub et al., 2006; Benekareddy et al., 2010; Weber and Andrade, 2010; Avesar and Gulledge, 2012), although previous work has demonstrated the capacity of serotonin and 5-HT_2A_ agonists to exert direct inhibitory effects through 5-HT_2A_ receptors or heteromers (Carr et al., 2002; Kurrasch-Orbaugh et al., 2003; González-Maeso et al., 2007; Moreno et al., 2011). We found that adding the selective 5-HT_2A_ antagonist MDL100907 abolished the remaining inhibitory effects elicited by 5-HT on the input–output of L6 neurons (no significant effects of 5-HT in WAY100635 and MDL100907: *F*_(1,32)_ = 0.8, *p* = 0.4, *n* = 5, repeated-measures two-way ANOVA; Fig. 2*C*). A similar blockade of the inhibitory effects of 5-HT on L6 neurons was also seen when other 5-HT_2A_ antagonists were applied together with WAY100635, such as ketanserin (2 µm) or ritanserin (300 nm to 1 µm; *F*_(1,48)_ = 0.3, *p* = 0.6, *n* = 7, repeated-measures two-way ANOVA).

To probe further the power of 5-HT_2A_ receptors to inhibit L6 pyramidal neurons in mPFC, we applied a potent 5-HT_2A_ agonist, TCB-2 (300 nm to 1 µM). Here, we observed a strong inhibition of L6 neuronal excitability, with a significant right-shift of the input–output relationship (inhibitory effect of TCB-2: *F*_(1,80)_ = 24, *p* < 0.0001, *n* = 11, repeated-measures two-way ANOVA; Fig. 2*D*). Pretreatment with MDL100907 abolished the inhibitory effect of TCB-2 (*F*_(1,16)_ = 1.2, *p* = 0.3, *n* = 3, repeated-measures two-way ANOVA). Taken together, our results suggest that 5-HT inhibition of mPFC L6 pyramidal neurons is mediated by a combination of 5-HT_1A_ and 5-HT_2A_ receptors acting in concert. However, substantial future work will be needed to elucidate the mechanisms by which these receptors individually and together work to suppress the excitability of L6 pyramidal neurons.

### Transgenic mouse for examining the effect of L6 activation on L5 interneurons

**Figure 3.**
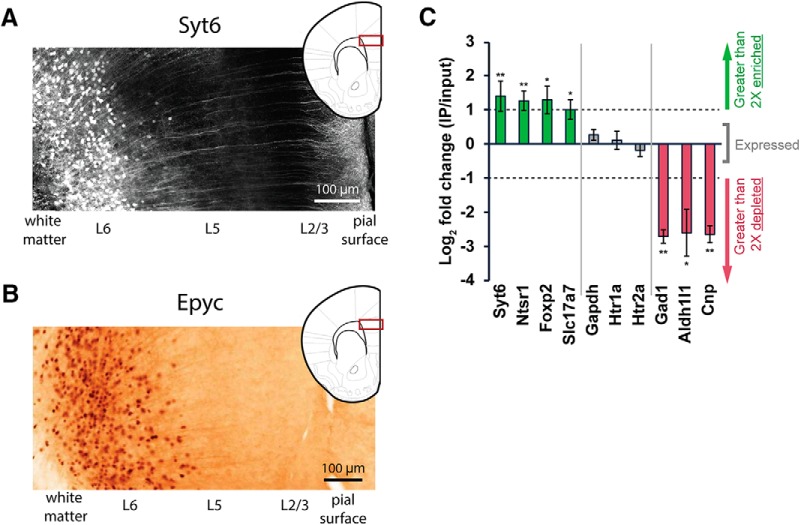
Characterization of L6 neurons in medial prefrontal cortex (mPFC) expressing synaptotagmin 6 and epiphycan. ***A***, Neurons expressing eGFP driven by the synaptotagmin-6 (Syt6) BAC promoter are localized to L6 pyramidal neurons in the prelimbic region of mPFC. ***B***, EGFP is also seen in L6 pyramidal neurons in prelimbic mPFC by anti-EGFP immunohistochemistry in Epyc-Cre mice crossed to a Cre-dependent eGFP reporter. Image is adapted from www.gensat.org. ***C***, Quantification (mean ± SEM) by qRT-PCR of the expression of selected genes in mPFC Epyc-vTRAP IP samples compared with whole PFC input. Positive values indicate enrichment in the IP, and negative values indicate depletion. Dotted lines indicate a twofold difference in either direction. Green bars, genes that are >2-fold enriched in the Epyc cells; red bars, genes that are >2-fold depleted; gray bars, genes expressed at levels similar to the rest of PFC. Of note, mRNAs for serotonin receptors *Htr1a* and *Htr2a* were expressed, but not enriched, in Epyc cells, a not-unexpected finding given the expression of 5-HT_1A_ and 5-HT_2A_ in other populations of neurons in mouse mPFC beyond L6. *p < 0.05; **p < 0.01 by Student’s *t* test.

It has been shown that L6 pyramidal neurons in primary sensory cortex exert robust gain modulation over superficial layers of the cortical column (Olsen et al., 2012) through strong connections to FS interneurons (Vélez-Fort and Margrie, 2012). In mPFC, recent work has demonstrated the importance of FS interneurons in L5 for performance on attention tasks (Kim et al., 2016). To investigate the effects of prefrontal L6 activation on its targets in the cortical column, we used the Epyc-Cre BAC transgenic mice that target Cre recombinase to L6 cells in mPFC, then generated Epyc-Cre;Ai:32 mice (Epyc-ChR2) to obtain expression of channelrhodopsin in those cells. Figure 3*A* and *B* shows a similar distribution of L6 cells labeled by Syt6-eGFP fluorescence, used in the initial electrophysiology experiments, and by Epyc-Cre, used for the optogenetic experiments. Because of the lack of reliable histological markers for L6 pyramidal neurons in mPFC, the TRAP technique (Heiman et al., 2008; Doyle et al., 2008) was used to interrogate the identity of the Epyc-Cre cells. An adeno-associated virus (AAV) vector (AAV-FLEX-EGFPL10a) to express EGFP-tagged ribosomal protein L10a (EGFPL10a) in a Cre-dependent manner was injected into the mPFC of Epyc-Cre mice, and anti-EGFP immunoprecipitations were performed to isolate tagged polysomes. Bound mRNAs were then purified and analyzed by qRT-PCR. These data are plotted in Figure 3*C*. There was a significant enrichment for the excitatory neuron marker, *Slc17a7* (VGluT1), in the Epyc TRAP IP compared with whole PFC input. Two genes known to be expressed in L6 corticothalamic cells, *Ntsr1* (Gong et al., 2007; Olsen et al., 2012; Mease et al., 2014) and *Foxp2* (Ferland et al., 2003), were also significantly enriched in IP samples. In contrast, genes that label inhibitory interneurons (*Gad1*), astrocytes (*Aldh1l1*), or oligodendrocytes (*Cnp*) were significantly depleted from the IPs. Taken together, these data suggest that Epyc-Cre labels a population of L6 corticothalamic pyramidal cells. Importantly, the qPCR also revealed that *Syt6* was highly enriched in the Epyc cells, demonstrating that the Syt6-eGFP and Epyc-Cre mice label an overlapping population of neurons. In contrast, levels of the housekeeping gene *Gapdh* were found to be similar between the TRAP IPs and whole PFC input. Similar results were obtained for the 5-HT receptors, *Htr1a* and *Htr2a,* suggesting these genes are expressed but not enriched in the Epyc cells, which was not surprising given the expression of 5-HT_1A_ and 5-HT_2A_ in other populations of neurons in mouse mPFC beyond L6 (Chalmers and Watson, 1991; Pompeiano et al., 1992, 1994; Cornea-Hébert et al., 1999; Amargós-Bosch et al., 2004).

### Optogenetic activation of L6 pyramidal neurons is sensitive to serotonin

**Figure 4.**
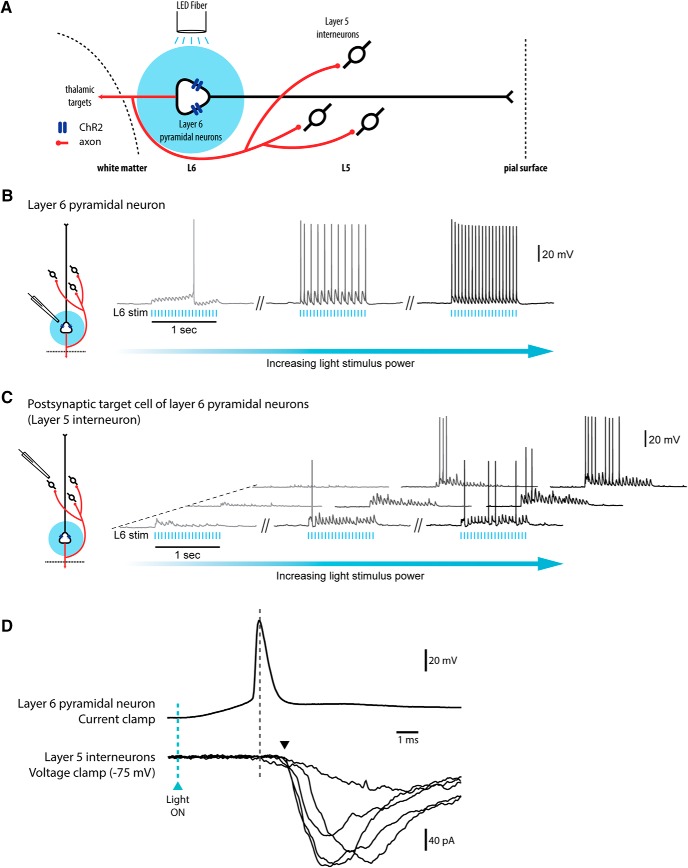
Optogenetic activation of L6 pyramidal neurons of medial prefrontal cortex excites L5 interneurons. ***A***, Schematic representation of light activation of L6 pyramidal neurons of medial prefrontal cortex in Epyc-ChR2 mice with axons projecting to L5 interneurons. ***B***, Channelrhodopsin-expressing pyramidal L6 neurons were robustly excited by targeted light stimulation over L6. The effects of increasing L6 light power are shown for one example L6 pyramidal neuron. ***C***, Light activation of L6 robustly excited L5 interneurons. The effects of increasing L6 light power are shown for three different L5 interneurons. ***D***, Top, close-up of the initial light-evoked action potential in L6 to show the timing from onset of light (blue dotted line) to peak of the spike (black dotted line). Scale bar: 20 mV, 1 ms. Note: In L6 the onset of depolarization from light is <1 ms. Bottom, voltage-clamp recording showing the light-evoked postsynaptic response in a L5 interneuron to demonstrate response latency. Scale bar: 40 pA, 1 ms. Postsynaptic responses in L5 interneurons were initiated 1.1 ± 0.3 ms after initial spike of L6 pyramidal neurons, as indicated by an arrow.

In electrophysiological experiments from Epyc-ChR2, we found that L6 pyramidal neurons, but not nonpyramidal neurons, were strongly depolarized upon light stimulation (473 nm, train of 2-ms-duration pulses at 20 Hz for 1 s), which was targeted to L6 mPFC with optic fiber (Fig. 4*A*, *B*). In contrast, prefrontal L6 neurons of littermate controls lacking channelrhodopsin did not respond to light stimulation. To verify that L6 pyramidal neurons were directly activated by light stimulation, we measured the kinetics of their light-evoked excitation. L6 pyramidal neurons rapidly responded to light (<1-ms latency to onset of excitation), consistent with direct activation through expressed channelrhodopsin (Ernst et al., 2008). This response rises to threshold, giving an action potential peak at 4.7 ± 0.7 ms (time to L6 spike; *n* = 5).

The channelrhodopsin-expressing L6 neurons from Epyc-ChR2 mice showed sensitivity to 5-HT similar to that of the Syt6-eGFP cells in the above experiments. Light-mediated excitation of Epyc-ChR2 L6 neurons was significantly suppressed in the presence of 5-HT (*F*_(1,24)_ = 10.3, *p* < 0.004, repeated-measures two-way ANOVA).

### Optogenetic activation of L6 drives excitation of L5 interneurons

Because fast-spiking GABAergic neurons in mPFC are important to normal performance in attention tasks (Kim et al., 2016), we patched mPFC L5 interneurons in Epyc-ChR2 mice as potential downstream projection targets of L6 pyramidal neurons. We anticipated that light-mediated activation of L6 pyramidal neurons by targeted optic fiber would elicit postsynaptic responses in patched L5 interneurons. GABAergic interneurons were visually identified by their morphology and intrinsic properties, and their spiking patterns in response to depolarizing current steps were documented. This experimental protocol yielded two distinct populations of interneurons: FS cells with characteristic action potential firing >40 Hz and nFS cells that displayed low-threshold firing characteristics. The intrinsic properties of these neurons are illustrated in Table 1. A subset of patched interneurons (*n* = 6 FS interneurons, *n* = 5 nFS interneurons) was filled with Alexa Fluor 594 (20 µm) or Texas red dextran (0.15%) in the patch solution to verify their morphology. Filled FS (6/6) and nFS (5/5) interneurons were morphologically characteristic of the respective subtypes of interneurons in cortex (Markram et al., 2004; Ascoli et al., 2008).

All of the L5 FS (*n* = 19) and nFS (*n* = 22) interneurons recorded responded to light stimulation positioned over L6 (20 Hz, 2- to 5-ms pulse duration, 20-pulse train). Light stimulation over other cortical layers did not produce a response. Latency between time to L6 spike and response onset in L5 interneurons was 1.1 ± 0.3 ms, consistent with a monosynaptic connection (Markram et al., 1997; Feldmeyer et al., 2005; Frick et al., 2008) from L6 (Fig. 4*C*). Activation of both FS and nFS interneurons by optogenetic stimulation of L6 was substantially and significantly reduced by TTX (*F*_(1,80)_ = 19, *p* < 0.0001, two-way ANOVA). Together with the need for light activation over L6, it appears that channelrhodopsin is predominantly localized in the L6 pyramidal cell bodies and not in axon terminals impinging on the L5 interneurons.

**Figure 5.**
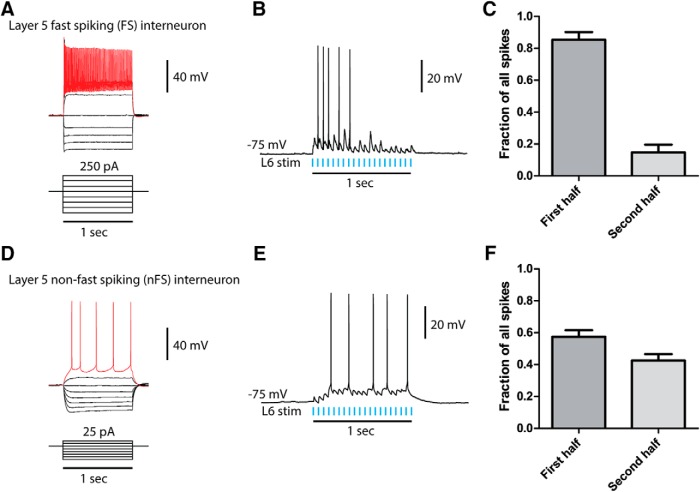
Two distinct groups of interneurons are found in L5 and are activated by light stimulation of L6 pyramidal neurons. ***A***, FS interneurons of L5 characterized by injection of current steps. ***B***, Representative trace of an L5 FS interneuron activated by L6. Note the rapidly depressing response to L6 activation. ***C***, Activation of FS interneurons by L6 elicited action potential firing primarily during the initial phase of activation that rapidly depressed over the duration of the stimulation (number of elicited action potentials in first half of stimulation vs. second half: *t*_9_ = 7.2, *p* < 0.0001, unpaired *t* test). ***D***, nFS interneurons of L5 characterized by injection of current steps. ***E***, Representative trace of a L5 nFS interneuron activated by L6, demonstrating a more regular firing pattern. ***F***, L5 nFS interneurons were activated by L6 and fired in a regular pattern over the course of the stimulation (number of elicited action potentials in first half of stimulation vs. second half: *t*_7_ = 1.9, *p* = 0.1, unpaired *t* test).

Light stimulation over L6 elicited action potential firing in 100% of FS cells (Fig. 4*D*) and 70% of nFS cells. The firing pattern elicited in these two types of interneurons was different, with a greater number of spikes seen at the start of L6 stimulation in FS neurons and a more evenly distributed firing pattern observed in the nFS neurons (Fig. 5). The minimal L6 light to elicit a suprathreshold excitatory response did not differ significantly between FS and nFS L5 interneurons (*t*_14_ = 0.2, *p* = 0.8, unpaired *t* test), despite a significant difference in input resistance (*t*_14_ = 4.3, *p* = 0.0006, unpaired *t* test; Table 1). In response to maximal L6 light stimulation of L6, FS interneurons fired more action potentials than nFS interneurons (*t*_14_ = 4.4, *p* = 0.0007, unpaired *t* test).

### Serotonin suppresses L6 activation of L5 interneurons

**Figure 6.**
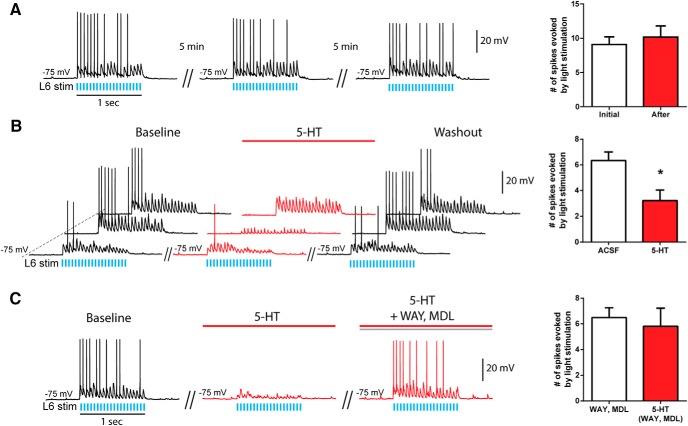
L6 activation of L5 interneurons in medial prefrontal cortex is stable over time but suppressed by 5-HT. ***A***, Excitatory effects on L5 interneurons by optogenetic activation of L6 were stable and repeatable over time. Shown here are the postsynaptic responses in a L5 interneuron to L6 light-stimulation repeated over 15 min. The number of spikes elicited initially and upon repetition in L5 interneurons is plotted on the bar graph at the right (mean ± SEM). There was no significant difference (*t*_11_ = 0.8, *p* = 0.4, paired *t* test), showing that the postsynaptic effect in L5 interneurons does not decrease over time under baseline conditions. ***B***, L6 activation of L5 FS interneurons was significantly suppressed by 5-HT (*t*_8_ = 3.8, *p* = 0.005, paired *t* test, *n* = 9). Shown here are repeated recordings (baseline, 5-HT, washout) from three different L5 interneurons. The number of spikes elicited at baseline and in the presence of 5-HT in L5 interneurons are illustrated in the bar graph on the right (mean ± SEM). ***C***, Antagonists of serotonergic 5-HT_1A_ and 5-HT_2A_ receptors blocked the inhibitory effects of 5-HT on L6 activation of L5 interneurons (*t*_15_ = 0.9, *p* = 0.4, paired *t* test, *n* = 16). Shown here is one representative L5 interneuron excited by L6 stimulation, which is suppressed by 5-HT applied alone, and no longer suppressed by 5-HT in the presence of WAY100635 and MDL100907. The results are plotted on the bar graph at the right (mean ± SEM).

Because optogenetic stimulation in L6 resulted in robust and highly stable excitation of interneurons in L5 that did not decrease over time at baseline conditions (*t*_11_ = 0.8, *p* = 0.4, paired *t* test; Fig. 6*A*), it was straightforward to test the effect of 5-HT on this local circuit. We found that 5-HT strongly and significantly suppressed the number of action potentials elicited in L5 interneurons by optogenetic activation of L6 (FS cells: *t*_8_ = 3.8, *p* = 0.005, *n* = 9, paired *t* test; nFS cells: *t*_6_ = 5.7, *p* = 0.001, *n* = 7, paired *t* test; Fig. 6*B*). Of note, this suppression appeared to arise from 5-HT effects in L6, since interneurons in L5 showed minimal direct responses to 5-HT at –75 mV (2.7 ± 6.0 pA, *p* = 0.9, *n* = 29, one-sample *t* test). Furthermore, these interneurons showed no change to spiking elicited by depolarizing steps of current amplitudes similar to those elicited by optogenetic stimulation of L6 (FS interneurons, *F*_(1,9)_ = 3.1, *p* = 0.1, repeated-measures two-way ANOVA; nFS interneurons, *F*_(1,18)_ = 3.4, *p* = 0.1, repeated-measures two-way ANOVA; data not shown). The excitation of L5 interneurons by L6 optogenetic activation was no longer sensitive to 5-HT upon blockade of 5-HT_1A_ and 5-HT_2A_ receptors (*t*_15_ = 0.9, *p* = 0.4, paired *t* test, *n* = 10 FS interneurons, *n* = 6 nFS interneurons; Fig. 6*C*). Overall, these results demonstrate the ability of L6 pyramidal neurons to excite a diverse group of inhibitory interneurons in L5 and the sensitivity of this effect to suppression by serotonergic 5-HT_1A_ and 5-HT_2A_ receptors.

## Discussion

In this study, we show robust serotonergic inhibition of L6 pyramidal neurons and their output to L5 interneurons. This suppression of L6 activity by 5-HT is driven by the combined effects of 5-HT_1A_ and 5-HT_2A_ receptors. Using transgenic mice and optogenetic techniques, we illustrate a functional link between L6 pyramidal neurons and L5 interneurons potentially important to performance on attention tasks. Light stimulation in L6 strongly excited L5 interneurons. This excitatory connection was inhibited by 5-HT and was restored in the presence of 5-HT_1A_ and 5-HT_2A_ antagonists. Taken together, these results suggest that 5-HT_1A_ and 5-HT_2A_ receptors mediate a strong inhibitory drive in L6 that can suppress its local activation of cortical targets in L5, which others have shown to be critical to attention (Kim et al., 2016).

### Prefrontal L6 pyramidal neurons excite a diverse group of interneurons in L5

We found that L6 pyramidal neurons excited both FS and nFS interneurons in L5 of mPFC. These groups of interneurons likely represent the parvalbumin-expressing (PV) and somatostatin-expressing (SOM) groups of interneurons, which together form the majority of interneurons in cortical L5 (Kawaguchi and Kubota, 1997; Rudy et al., 2011). Both perisomatic PV and dendrite-targeting SOM interneurons are strong mediators of activity on downstream cortical pyramidal output (Kawaguchi and Kubota, 1997; Glickfeld et al., 2009; Kvitsiani et al., 2013; Hangya et al., 2014), with mPFC PV interneuron activity particularly important to normal performance on attention tasks (Kim et al., 2016). Our finding of a functional connection between L6 and L5 interneurons suggests a means by which L6 could influence mPFC cortical gain modulation, as has been observed in primary sensory cortices (Olsen et al., 2012; Bortone et al., 2014). These findings are in agreement with previous work in primary sensory cortex that examined anatomical and functional connections within the cortical column (Zhang and Deschênes, 1997; Thomson et al., 2002; Mercer et al., 2005; Watts and Thomson, 2005; West et al., 2006; Kim et al., 2014). This research suggests that activation of L6 is a driver of intracortical inhibition leading to a widespread suppression of cortical targets, observed *in vivo* in visual cortex (Beierlein et al., 2003; Olsen et al., 2012; Bortone et al., 2014). The L6 excitation of nFS in addition to FS interneurons in mPFC suggests additional complexity in association cortex. Our data show for the first time that excitatory output from L6 can drive interneuron activity in L5 in mPFC, a region critical for attention and other executive functions.

### Serotonergic inhibition of this L6-to-L5 intracortical circuit

We found that 5-HT, via stimulation of 5-HT_1A_ and 5-HT_2A_ receptors, inhibited L6 pyramidal neurons and their activation of L5 interneurons. These two receptors show substantial colocalization in L6 pyramidal neurons in mPFC of mouse (Table 3 in Amargós-Bosch et al., 2004), yet typically appear to exert opposing electrophysiological effects in other cortical layers (Benekareddy et al., 2010; Avesar and Gulledge, 2012; Stephens et al., 2014). Whereas 5-HT_1A_ receptors are known inhibitory receptors acting via Kir3 channels (Goodfellow et al., 2014; Johnston et al., 2014), 5-HT_2A_ receptors act through a less well-characterized set of channels to excite certain populations of neurons, including a subset of L5 pyramidal neurons in mPFC (Willins et al., 1999; Benekareddy et al., 2010; Weber and Andrade, 2010; Avesar and Gulledge, 2012). Direct inhibition of L6 pyramidal neurons by 5-HT_2A_ receptors could arise from a number of possible mechanisms, including the suppression of sodium channels (Carr et al., 2002) or via 5-HT_2A_ heteromers with inhibitory signaling (González-Maeso et al., 2007; Moreno et al., 2011; Viñals et al., 2015). Pyramidal neurons in L6 also have prominent afterhyperpolarizations (Proulx et al., 2015), known to affect excitability. Accordingly, the Gα_q_-coupled 5-HT_2A_ receptors may affect the excitability of these neurons by modulating channels contributing to different phases of the afterhyperpolarization (Gulledge and Stuart, 2005; Gulledge et al., 2009; but see also: Villalobos et al., 2005; Villalobos et al., 2011). Complex and carefully controlled future work will be necessary to identify the mechanisms underlying the 5-HT_2A_ receptor–mediated inhibition of L6 pyramidal neuron excitability.

In investigating FS and nFS interneurons in L5, we found that the majority of these cells did not respond strongly to 5-HT. A minority showed electrophysiological responses (FS, 4/13; nFS, 2/16), predominantly inward currents (less than –20 pA) that were insufficient to elicit spiking. These proportions are consistent with the literature on the expression of 5-HT receptors only in a small proportion of L5 interneurons (Abi-Saab et al., 1999; Santana et al., 2004; Rudy et al., 2011; Celada et al., 2013). Our findings were not significantly altered by the inclusion or exclusion of these neurons. Control experiments with GABA-A and GABA-B blockers suggest that 5-HT receptors on interneurons are not significantly involved in the 5-HT suppression of L6 pyramidal neurons. Taken together, our data support the hypothesis that the combined activation of both 5-HT_1A_ and 5-HT_2A_ receptors can inhibit neuronal excitability in L6 neurons of prefrontal cortex. However, further pharmacological work is required to examine the specific downstream mechanisms underlying this inhibition of L6 pyramidal neurons. Furthermore, additional investigations into the consequences of serotonergic inhibition of L6 on local network dynamics will provide more insight into the nature of these important associative circuits and how they control attention.

### Serotonin, prefrontal attention circuitry, and attention deficits in psychiatric illness

Prefrontal attention circuitry is complex, and attentional performance can be perturbed by extremes of mPFC activity in either direction (Pezze et al., 2014). Serotonin shapes and biases attention in humans and rodents, with low levels of 5-HT enhancing attention (Schmitt et al., 2000; Gallagher et al., 2003; Booij et al., 2005) and higher levels of 5-HT disrupting attention (Riedel et al., 2005; Wingen et al., 2007; Watson et al., 2015) through 5-HT_1A_ and 5-HT_2A_ receptors (Wingen et al., 2007). Stress is well known to raise prefrontal serotonin levels (Adell et al., 1997; Fujino et al., 2002; Bland et al., 2004), and similar behavioral manipulations disrupt attention (Minor et al., 1984; Sänger et al., 2014). Intriguingly, elevation of intracortical 5-HT has been strongly associated with deficits in attention (Puumala and Sirviö, 1998) and increases in impulsivity (Dalley et al., 2002). Specific activation of 5-HT_1A_ and 5-HT_2A_ receptors results in similar attention deficits (Carli and Samanin, 2000; Koskinen et al., 2000). Conversely, infusion of antagonists to 5-HT_2A_ receptors into mPFC improved performance on attention tasks in rodents and reduced impulsivity (Passetti et al., 2003; Winstanley et al., 2003).

Although manipulation of 5-HT receptors specifically in mPFC can manipulate attention, certain key experiments (e.g., attention under stress or in models of dysregulated 5-HT signaling) remain to be done. Based on our findings, rising levels of cortical 5-HT may increase cortical noise owing to suppression of L6-mediated cortical inhibition. Increasing the signal-to-noise ratio is strongly correlated with attentional focus (Briggs et al., 2013; Pratte et al., 2013), whereas deficits can arise from increasing cortical noise in prefrontal cortex (Pezze et al., 2014), Similarly, disruptions to normal excitation of L6 neurons of mPFC can lead to attention deficits in rodents, although previous studies have focused on cholinergic stimulation of L6 (Bailey et al., 2010; Guillem et al., 2011). Our study is the first to demonstrate the strong inhibitory modulation exerted on L6 by 5-HT and the resultant decrease in its ability to stimulate interneuron activity in L5. Taken together, this evidence shows that L6 of mPFC is a candidate locus of action for the modulatory effects of 5-HT on attention. Based on recent work in nonhuman primates (Watson et al., 2015), it is tempting to speculate that 5-HT levels in deep mPFC may modulate the balance between social vigilance and attentional task performance, a phenomenon that is impaired in several neuropsychiatric illnesses.

## Synthesis

The decision was a result of the Reviewing Editor Douglas Bayliss and the peer reviewers coming together and discussing their recommendations until a consensus was reached. A fact-based synthesis statement explaining their decision and outlining what is needed to prepare a revision is listed below. The following reviewers agreed to reveal their identity: Vikaas Sohal, Robert Foehring

Two reviewers have assessed this report, and they concur that the paper provides interesting and important new information regarding serotonin effects in the prefrontal cortex. Although generally pleased with the study, they raise a few concerns that should be addressed in a revised manuscript. Specifically, experiments with a suitable agonist should be included to further verify responses attributed to 5-HT1A receptors. In addition, caveats and interpretive issues with respect to potential for misidentification of regular spiking cells as interneurons should be explicitly acknowledged and discussed, and some evaluation of the presence and 5-HT modulation of a sAHP in L6 neurons should be addressed. It was also suggested that the visual abstract could be improved. The comments from both reviewers are provided herewith.

Reviewer #1

Advances the Field

The manuscript describes a new effect of 5-HT to hyperpolarize L6 pyramidal neurons.

Visual Abstract

The visual abstract is a bit too confusing -- I don't think it's necessary to address optogenetic stimulation here, and I think something showing the receptors involved would be helpful.

Comments to the Authors

Overall this is a straightforward and nice work. My major comment is that the pharmacology of serotonin receptors is tricky and as a result, it is standard practice to do experiments with both agonists and antagonists. Since the main effect is driven in large part by 5-HT1A receptors it seems like a necessary experiment is to verify that the effect can be reproduced using a 5-HT1A agonist, e.g., 8-OH-DPAT.

Reviewer #2

Advances the Field

The experiments are elegant and address an important topic: the actions of 5HT on cells and circuits in PFC that have been implicated in attentional mechanisms. The findings are novel in that actions of 5HT2A receptors in L6 cells appears to be quite different than actions of 5HT on pyramidal neurons from other layers.

Visual Abstract

The visual abstract illustrates the optogenetic approach used and circuit addressed in the MS.

Comments to the Authors

This manuscript addresses an important topic: the actions of 5HT on neurons in medial PFC that have been implicated in attention tasks. The authors use an impressive array of techniques to address the actions of 5HT on L6 pyramidal neurons in prefrontal cortex of mice. A main finding was that bath applied 5HT hyperpolarizes L6 pyramidal neurons (outward current in VC) through activation of both 5HT1A and 5HT2A receptors. The same two receptors mediate a reduction in firing rate and gain reduction. The effects of 5HT2A activation are novel and surprising, given that in L2/3 and L5, 5HT2A receptor activation causes depolarization and firing increases. The authors generated a mouse with ChR expression selectively in L6 pyramidal cells. This mouse was nicely characterized with IP experiments. This mouse was used to activate L6 neurons to spiking with light. The 5HT effects were verified in L6 neurons and recordings were made from small, L5 neurons to characterize direct synaptic activation of the L5 cells by photoactivation of L6 pyramidal neurons. These experiments revealed monosynaptic (by delay time) excitation from L6 pyramids to the L5 cells. The L5 cells were classified as FS interneurons and non-FS interneurons by physiology and small size and round soma. 5HT suppressed L6 activation of both of these cell types. The findings are significant, data are of high quality and the manuscript is well-written.

Major:

I have two main criticisms.

1. The first regards the conclusion that all of the L6 projections to L5 that they are studying are to GABAergic interneurons. I do not dispute the FS cells as they have been shown repeatedly to be a subset of GABAergic interneurons. It is also true that there are many non-FS interneurons in L5, but there are also small pyramidal cells in that layer as well. None of the criteria described would differentiate between a regular spiking pyramidal cell and a regular spiking interneuron. The authors should refer to these cells as regular spiking (RS) and consider that some of them may be excitatory when discussing the implications of the connections and actions of 5HT (e.g., page 18). If not all cells are GABAergic, the significance of the discussion is reduced. The authors mention a subset of cells with an Alexa-dye or Texas red included in the pipet (page 15). For both FS and RS cells, how many cells (out of how many) were filled in this way? What percentage of them were definitively interneurons? Non pyramidal cells identified in this way could safely be deemed interneurons.

2. My other criticism regards the 5HT / 5HT2A effects on firing rate and gain in L6 pyramidal neurons (Figure 2). The authors mention that in other regions and cortical layers, 5HT2A activation increases firing rate and gain. In those cells, 5HT2A activation reduces the sAHP and underlying conductance, thus reducing spike frequency adaptation and increasing firing. In the present study, did L6 cells express a sAHP and if so, was it modulated by 5HT (and especially 5HT2A)?

Minor comments:

1. Table 1: was the liquid junction potential corrected for these cells? What is the LJP (was it measured)? The membrane potentials are very hyperpolarized.

2. Table 1: How was AP amplitude measured? The amplitudes do not appear to overshoot 0 mV if both RMP and AP amplitudes are correct?

3. Page 12, lines 272-278: How many cells were tested in each of these groups?

4. Page 17, lines 375-379: These data should be indicated as "not shown".
